# A Hydroxynaphthol Blue-Based Loop-Mediated Isothermal Amplification Assay for Closed-Tube Detection of the Streptomycin Resistance Gene *aadA1* in *Salmonella*

**DOI:** 10.3390/vetsci12111094

**Published:** 2025-11-17

**Authors:** Yuxiang Shen, Yeqing Zheng, Meiquan Li, Yanli Du, Heng Yang, Fangjie Li, Bin Wang, Xiao Wang

**Affiliations:** 1 Engineering Research Center for Novel Livestock and Poultry Vaccines and Key Industrial Technologies of Yunnan Provincial Department of Education, Kunming University, Kunming 650214, China; shenyuxiang59@gmail.com (Y.S.); wangbbiomed@kmu.edu.cn (B.W.); 2 School of Medicine, Kunming University, Kunming 650214, China; 3 College of Agriculture and Life Sciences, Kunming University, Kunming 650214, China; zhengyeqing07@163.com (Y.Z.); limeiquan2010@163.com (M.L.); yanli@kmu.edu.cn (Y.D.); yangheng2008.cool@163.com (H.Y.); 15892697014@163.com (F.L.)

**Keywords:** loop-mediated isothermal amplification, streptomycin resistance gene (*aadA1*), *Salmonella*, hydroxynaphthol blue

## Abstract

The *aadA1* gene, which confers resistance to streptomycin, is typically located within class Ⅰ integrons. This genetic context enables its dissemination among diverse Gram-negative bacteria, such as *Salmonella*, and facilitates its potential transfer to humans through the food chain or into agricultural environments via manure. Hence, the detection of *aadA1* genes is crucial for surveillance, understanding transmission dynamics, and informing strategies to mitigate the spread of resistant bacteria. Conventional *aadA1* detection relies on time-consuming or equipment-intensive molecular assays like PCR or qPCR. In this study, we developed and optimized a closed-tube, hydroxynaphthol blue (HNB) -based loop-mediated isothermal amplification (LAMP) assay to detect *Salmonella aadA1* gene and performed evaluation and validation against conventional PCR. The LAMP assay demonstrated high specificity and sensitivity, with a detection limit of 1 pg (190 copies) of genomic DNA per reaction, which is tenfold higher than that of conventional PCR. In parallel testing of 40 *Salmonella* DNA samples, the optimized LAMP assay achieved a detection rate of 100.0% for the *aadA1* gene in streptomycin-resistant isolates, compared to 96.3% by conventional PCR. Among the streptomycin-susceptible isolates, the LAMP assay also showed a higher detection rate (38.5%) for the *aadA1* gene, compared to 23.1% by conventional PCR. Consequently, the LAMP assay developed in this study for detecting the *aadA1* gene offers a combination of simplicity, speed, visual readout, high specificity, and sensitivity, making it particularly suitable for rapid field detection in antimicrobial resistance surveillance programs.

## Simple Summary

Antibiotic resistance genes can spread to humans through the food chain or animal waste, posing a serious global health threat. One such gene, called *aadA1*, is commonly found in *Salmonella* and make it resistant to the antibiotic streptomycin. Detecting this gene is essential for monitoring its spread and guiding appropriate antibiotic use. In this study, we developed a simple, rapid, and low-cost molecular method—known as loop-mediated isothermal amplification (LAMP)—to detect the *aadA1* gene. The test only requires a constant temperature of 64 °C, and a dye (hydroxynaphthol blue) is added to the reaction tube to indicate the result by a clear color change from violet to sky blue. The method can detect whether *Salmonella* carries the *aadA1* gene in as little as 35 min, with a detection limit as low as 1 pg (190 copies) of bacterial genome DNA. When testing 40 clinical *Salmonella* strains, the LAMP method showed a higher detection rate than conventional PCR. This technique provides a practical tool for regions with limited resources and holds promise for widespread use in local laboratories.

## 1. Introduction

The horizontal transfer of antimicrobial resistance genes (ARGs) via mobile genetic elements (MGEs)—such as plasmids, integrons, transposons, and integrative and conjugative elements—is a key mechanism driving the dissemination of resistance [1,2,3]. This mobility poses a substantial threat, as exemplified by the *aadA1* gene, which confers resistance to streptomycin [4]. Of particular concern is the association of *aadA1* with class I integrons, allowing it to spread efficiently among Gram-negative bacteria, including *Salmonella* [5,6]. As a sentinel gene often linked with other resistance determinants on MGEs, monitoring *aadA1* provides an early warning for the dissemination of multidrug resistance. As a major foodborne pathogen, *Salmonella* plays a dual role: it causes human disease and acts as a vehicle for ARG dissemination. The acquisition of *aadA1* by *Salmonella* in food animal reservoirs, followed by potential transmission through the food chain and environment via contaminated manure, highlights a critical risk pathway [7,8,9]. Consequently, monitoring *aadA1* in *Salmonella* is essential for tracking resistance and mapping the flow of MGEs, offering a strategic approach to mitigating a multifaceted public health threat.

PCR, multiplex PCR, and qPCR are commonly used for the detection of *aadA1* genes [5,10,11]. However, these methods possess inherent limitations that restrict their utility in resource-limited or point-of-care settings. Conventional PCR and multiplex PCR require post-amplification gel electrophoresis for product visualization, which is time-consuming, labor-intensive, and increases the risk of amplicon contamination. Although qPCR eliminates the need for gel electrophoresis and allows for quantitative analysis, it remains dependent on sophisticated and costly thermal cycling equipment, as well as fluorescent probes or DNA-binding dyes, which elevate the overall cost per reaction [12]. These limitations collectively hinder large-scale, field-friendly monitoring of *aadA1* at critical points like farms and local clinics, creating a pressing need for a simple, rapid, cost-effective, and equipment-independent detection method deployable outside central laboratories.

Loop-mediated isothermal amplification (LAMP) is a nucleic acid amplification technique that employs four to six specific primers to recognize six to eight distinct regions of the target DNA under isothermal conditions (typically 58 °C to 65 °C), enabling rapid amplification within 60 min [13]. Amplification products can be assessed through real-time monitoring of turbidity or endpoint colorimetric detection using metal-ion indicators such as hydroxynaphthol blue (HNB) or intercalating fluorescent dyes [14,15,16]. Known for its high sensitivity and specificity, the LAMP assay is particularly advantageous for deployment in resource-limited settings, as it requires minimal instrumentation and technical expertise, thereby offering a cost-effective diagnostic alternative [17]. While LAMP has been widely used to detect various infectious pathogens and certain antibiotic resistance genes—including *sul1*, *sul2*, *sul3*, and *msrA*—its application for monitoring the streptomycin resistance gene *aadA1* remains limited [18,19,20,21]. Given the role of *aadA1* as a mobile genetic element-borne sentinel gene frequently associated with multidrug resistance in *Salmonella* and other enteric pathogens, there is a clear and pressing need to develop a dedicated LAMP assay for its rapid and field-deployable detection.

In this study, we describe the development of a LAMP for the rapid, specific, and sensitive detection of the *aadA1* and its use for the rapid screening of clinical isolates for *aadA1*.

## 2. Materials and Methods

### 2.1. Salmonella Strains and Antimicrobial Susceptibility Testing

The *Salmonella* reference strains used in this study were isolated and maintained in our laboratory. The serotype of each reference strain was determined using a glass slide agglutination test with commercially available *Salmonella* Antisera (Ningbo Tianrun China, Ningbo, China), following the White–Kauffmann–Le Minor scheme. The presence or absence of the *aadA1* gene in these strains was confirmed by Sanger sequencing of the PCR amplicon using specific primers [22]. Based on these results, five *aadA1*-positive and five *aadA1*-negative reference strains were selected for this study (Table 1). The *Salmonella* strain LLC-35, confirmed to carry the *aadA1* gene, served as the source of genomic DNA for the development and optimization of the LAMP assay (Table 1). A total of 40 clinical *Salmonella* isolates were obtained from diseased poultry in China and were confirmed by standard bacteriological methods coupled with PCR amplification and sequencing of the genus-specific *invA* gene [23].

For phenotypic resistance analysis, antimicrobial susceptibility testing (AST) for streptomycin was performed on all strains mentioned above (including the 10 reference strains and 40 clinical isolates) using the disk diffusion method, in strict accordance with CLSI guidelines (M100, 2025). *Escherichia coli* ATCC 25922 was used for quality control of the AST procedure.

### 2.2. DNA Extraction

The purified bacteria were inoculated into Luria–Bertani (LB) broth (Coolaber, Beijing, China) for overnight incubation at 37 °C. Then, a rapid DNA isolation kit (Omega Bio-Tek, Norcross, GA, USA) was used to extract bacterial DNA according to the manufacturer’s instructions. The purity and concentration of the DNA were determined using a BioDrop μLite spectrophotometer (Biochrom, Cambridge, UK).

### 2.3. Design of the LAMP Primers

The LAMP primers were designed based on the *aadA1* reference sequence (GenBank accession no. GQ924769.1) using the online NEB LAMP Primer Design Tool V1.5.1 (https://lamp.neb.com/#!/, accessed on 18 March 2025). The resulting primers, including outer primers (F3 and B3), inner primers (FIP and BIP), and the loop primer (LB), are listed in Table 2. The conventional PCR primers used for the *aadA1* gene and the reference are also provided in Table 2.

### 2.4. Optimized LAMP Assay

The LAMP assay protocol was optimized with a set of five primers designed in this study, while the reaction components and conditions for closed-tube visual detection were simultaneously investigated. The optimization process involved systematically testing different concentrations of MgSO_4_, dNTPs, and Bst DNA polymerase, different primer set ratios (inner:outer:loop), as well as different reaction temperatures and incubation durations. The optimized parameters are summarized in Table 3. The basal reaction mixture contained 10 × ThermoPol Buffer, 8 mM MgSO_4_, 1.2 mM each of dNTPs, 8 U Bst DNA polymerase (Vazyme, Nanjing, China), 1 M betaine (Beyotime, Shanghai, China), and five primers (FIP/BIP at 0.8 μM, F3/B3 at 0.2 μM, LB at 0.4 μM). Nuclease-free water was utilized to make up the total volume of 25 μL. Reaction components were initially mixed and aliquoted to separate tubes, followed by addition of 1 μL DNA template. Reaction tubes were subjected to isothermal amplification at 60 °C for 55 min, then enzyme deactivation at 80 °C for 2 min using a thermocycler. After amplification, the results were initially observed visually via the HNB color change. Theoretically, a positive reaction is indicated by a sky blue color, whereas a negative result is shown as violet. To objectively quantify this change, the reaction mixture was subjected to UV-Vis spectrophotometric analysis. Briefly, the 2 µL reaction product was scanned from 400 to 700 nm, with a shift in the absorption peak to ~650 nm serving as a quantitative indicator of a positive result. Additionally, product specificity was confirmed by standard agarose gel electrophoresis of a 5 µL aliquot. The gel image was then converted to a grayscale representation using Adobe Photoshop CS6.

### 2.5. Specificity and Sensitivity of the LAMP Assay

The specificity of the LAMP assay was developed by testing five reference strains with known *aadA1* genotypes and five *aadA1*-free reference strains. The shortest band from the LAMP products after gel electrophoresis was purified using a gel extraction kit (Omega Bio-Tek, Norcross, GA, USA). The purified product was subsequently subjected to TA cloning (TransGen, Beijing, China), and positive clones were sent to Tsingke Biotechnology Co., Ltd. (Beijing, Chian) for sequencing.

The sensitivity of the LAMP assay was assessed using tenfold serial dilutions of genomic DNA extracted from the *Salmonella* LLC-35 strain, with concentrations ranging from 10 ng/µL to 0.1 pg/µL (corresponding to 1.9 × 10^6^ to 1.9 × 10^1^ copies/μL). The diluted samples were amplified simultaneously by the optimized LAMP method and conventional PCR (targeting a 489 bp fragment of *aadA1*) for a direct comparison of their detection sensitivities.

### 2.6. Parallel Detection of Clinical Isolates by LAMP and PCR Assays

Genomic DNA was extracted from all 40 clinical *Salmonella* isolates. The presence of the *aadA1* gene was subsequently screened using both the visual LAMP assay developed in this study and conventional PCR. In parallel, antibiotic sensitivity test for streptomycin resistance—consistent with the resistance profile conferred by *aadA1*—was performed on all 40 isolates. Strains showing discordant results between phenotypic and molecular methods were further validated by Sanger sequencing, which was conducted by Tsingke Biotechnology Co., Ltd.

### 2.7. Statistical Analysis

To ensure reproducibility, the assay was rigorously evaluated through three independent technical replicates. The results expressed as mean ± standard deviation (SD).

## 3. Results

### 3.1. Optimization of LAMP Reaction Component Concentrations

To enhance the accuracy of the LAMP assay, optimization was performed on the concentrations of MgSO_4_, dNTPs, Bst DNA polymerase, and the ratio of primers. The reaction products were subjected to UV-Vis spectrophotometric scanning from 400 to 700 nm, which consistently revealed two characteristic absorption peaks at approximately 590 nm and 650 nm, corresponding to the violet and sky-blue colors, respectively.

**Figure 1:**
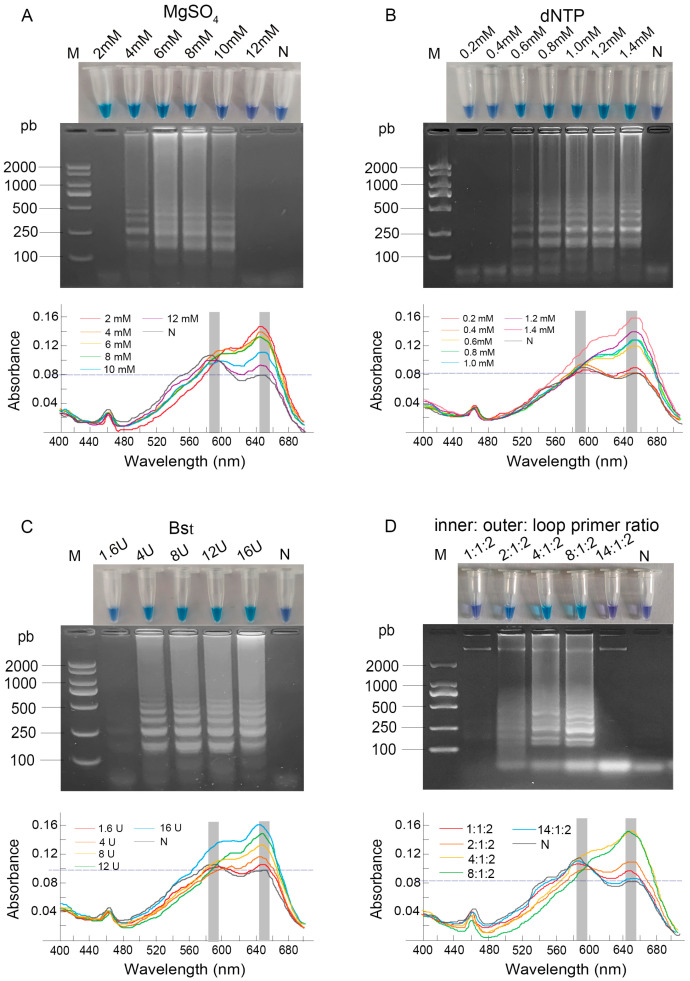
Optimization of LAMP reaction component concentrations. (**A**) MgSO_4_ (2–12 mM); (**B**) dNTP (0.2–1.4 mM); (**C**) Bst DNA polymerase (1.6–16 U); (**D**) inner: outer: loop primer ratio (1:1:2–14:1:2). Results were analyzed by direct visual observation of tube color (**upper panel**), agarose gel electrophoresis (**middle panel**), and UV-Vis spectrophotometric analysis (**lower panel**). Lane M: DL2000 DNA Marker; Lane N: negative control; concentrations for all other lanes are as labeled on the figure.

As the MgSO_4_ concentration increased, the reaction color shifted from sky blue to violet. Spectrophotometric analysis confirmed this transition as a systematic shift in the dominant absorption peak from 650 nm to 590 nm. Distinct ladder-like bands were only observed at concentrations of 4–10 mM (Figure 1A). Consequently, based on the combination of a definitive color change, a dominant peak at 650 nm, and robust amplification, 6 mM was selected as the optimal MgSO_4_ concentration.

When the dNTP concentration was increased from 0.6 to 1.4 mM, the post-amplification color of the products gradually transitioned from dark blue to sky blue. This visual change was quantitatively supported by a steady increase in the absorbance value at 650 nm. The electrophoretic results were consistent with the visual and spectrophotometric observations. The brightest bands, indicating the most complete amplification, along with the highest A_650_ value, were observed at dNTP concentrations of 1.4 mM (Figure 1B). Consequently, 1.4 mM was chosen as the optimal dNTP concentration.

Regarding the amount of Bst DNA polymerase, the reaction with 1.6 U produced a dark blue color, which corresponded to absorption peaks of nearly equal height at both 590 nm and 650 nm, indicating an intermediate state. In contrast, reactions with 8 U, 12 U, and 16 U exhibited a sky blue color with a progressively higher and dominant absorption peak at 650 nm, demonstrating a clear enhancement of amplification with increasing enzyme amounts. The electrophoretic results corroborated the visual findings (Figure 1C). Taking into account both the experimental results and cost-effectiveness, 8 U was identified as the optimal amount of Bst DNA polymerase.

The primer ratio (inner:outer:loop) critically influenced the reaction outcome. Ratios of 1:1:2 and 14:1:2 yielded violet products exhibiting a dominant 590 nm peak, whereas 4:1:2 and 8:1:2 produced sky blue products with a pronounced 650 nm peak. Electrophoretic analysis confirmed the most complete amplification at the 8:1:2 ratio, which also showed the most distinct spectral profile of a positive reaction (Figure 1D), and it was consequently selected as optimal.

### 3.2. Optimization of LAMP Conditions

**Figure 2:**
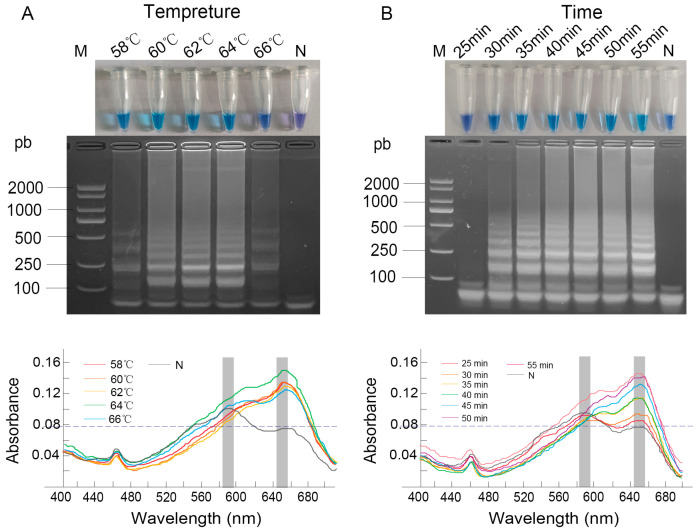
Optimization of LAMP reaction conditions. (**A**) Temperature gradient (58–66 °C); (**B**) Reaction time. Results were analyzed by direct visual observation of tube color (**upper panel**), agarose gel electrophoresis (**middle panel**), and UV-Vis spectrophotometric analysis (**lower panel**). Lane M: DL2000 DNA Marker; Lane N: negative control; conditions for all other lanes are as labeled in the figure.

The optimization of reaction conditions involved both temperature and time. When the reaction temperature was between 60 °C and 64 °C, the product exhibited a sky-blue color, while at 58 °C and 66 °C, it turned dark blue. UV-Vis spectrophotometric analysis provided quantitative support for these observations, revealing that the absorbance at 650 nm reached approximately 0.16 at 64 °C, higher than the values around 0.14 at 58–62 °C and 0.13 at 66 °C. Electrophoretic analysis confirmed amplification across the entire temperature range of 58 °C to 66 °C (Figure 2A). Based on the principle that higher temperatures generally promote greater amplification specificity, along with the most favorable spectral profile, 64 °C was selected as the optimal reaction temperature.

For reaction time, a positive sky-blue color and distinct ladder-like bands were consistently observed from 35 to 55 min. Spectrophotometric monitoring showed a clear increase in the absorbance at 650 nm over time, with a notable rise observed starting from 35 min, at which point the A_650_ value became significantly higher than the A_590_, confirming efficient amplification (Figure 2B). The shortest time of 35 min was chosen as optimal to ensure assay rapidity while maintaining robust amplification.

### 3.3. Optimization of HNB Concentration for the LAMP Assay

**Figure 3:**
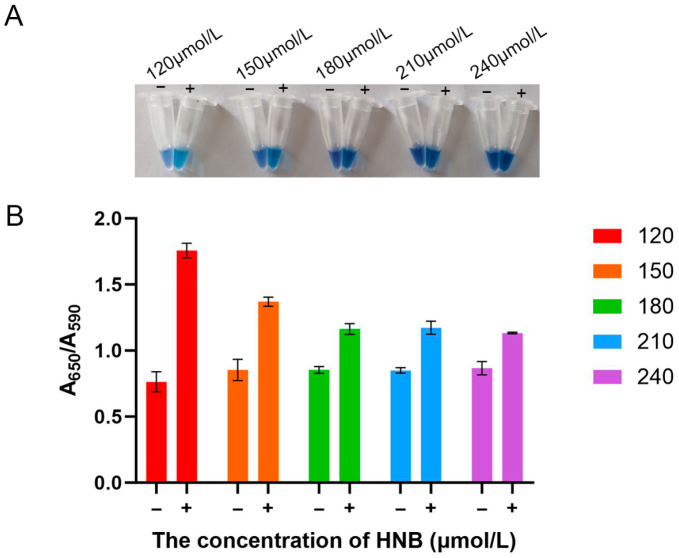
Optimization of hydroxynaphthol blue (HNB) concentration for the LAMP assay. (**A**) Visual assessment of color change in reaction tubes across the tested concentration range (120–240 μmol/L). (**B**) Quantitative analysis of the colorimetric reaction. “−“, negative control; “+”, positive control.

HNB, a metallochromic indicator, detects amplification products through a distinct color change before and after the reaction. Visually, 120 μmol/L HNB produced the most distinct color contrast between positive (sky blue) and negative (violet) samples (Figure 3A). This observation was quantitatively confirmed by measuring the A_650_/A_590_ ratio, where a higher value indicates more complete amplification. As shown in Figure 3B, while negative controls maintained a stable baseline ratio of approximately 0.8 across all concentrations, the positive reactions reached a peak A_650_/A_590_ value of 1.7 at 120 μmol/L, with the ratio progressively decreasing at higher concentrations. Therefore, 120 μmol/L HNB was selected as the optimal concentration, based on both the clearest visual readout and the strongest supporting spectrophotometric evidence.

### 3.4. Specificity of the Optimized LAMP Assay for aadA1 Gene

**Figure 4:**
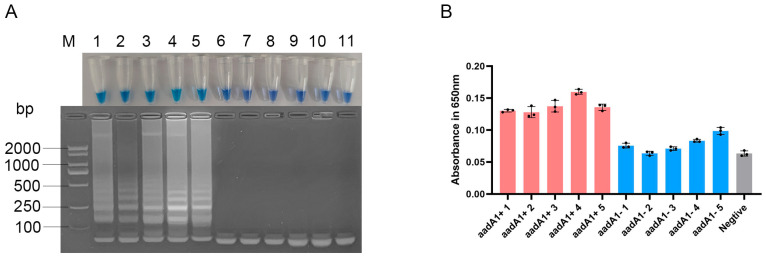
Specificity of the optimized LAMP assay for *aadA1* gene. (**A**) Visualization by tube color (**top**) and gel electrophoresis (**bottom**). (**B**) quantitative evaluation of colorimetric LAMP products by spectroscopic evaluation at 650 nm. Lane M: DL2000 DNA Marker; Lanes 1–5: *aadA1*+ *Salmonella* strains; Lanes 6–10: *aadA1*− *Salmonella* strains; Lane 11: negative control; Pink bars: *aadA1*+ *Salmonella* strains; Blue bars: *aadA1*− *Salmonella* strains; Gray bar: negative control.

The LAMP assay successfully amplified all the reference *Salmonella* strains known to have *aadA1* but were negative for the *aadA1*-free reference strains (Figure 4A). This specificity was quantitatively confirmed by measuring the absorbance at 650 nm, where products from *aadA1*-positive strains yielded values consistently greater than 0.12, whereas those from negative strains remained below 0.10 (Figure 4B). These spectrophotometric results were in complete agreement with both the visual color change and agarose gel electrophoresis. Furthermore, sequencing of the shortest bands of the LAMP product confirmed that the amplified sequences were 100% identical to the *aadA1* gene, unequivocally verifying the assay’s specificity.

### 3.5. Sensitivity of the Optimized LAMP Assay for aadA1 Gene Against PCR

**Figure 5:**
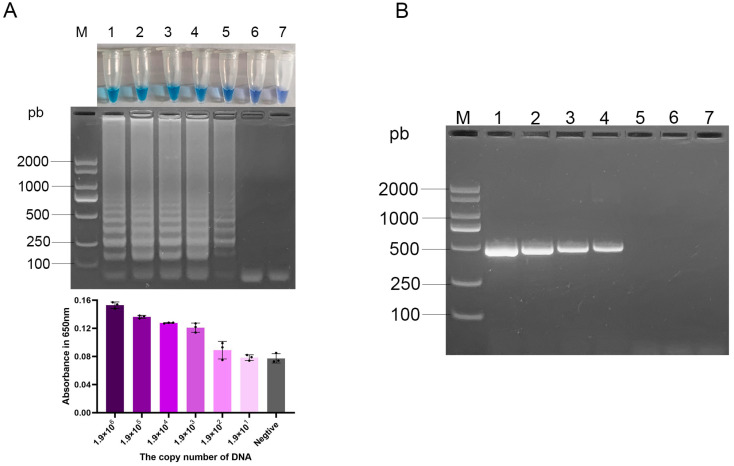
Sensitivity of the optimized *aadA1* LAMP and PCR assays to serial DNA dilution. (**A**) Visualization of LAMP results: tube color change (**top**), agarose gel electrophoresis (**middle**), and corresponding absorbance values at 650 nm ((**bottom**), bar chart represents mean ± SD); (**B**) PCR results by gel electrophoresis. Lane M: DL2000 DNA Marker; Lanes 1–6: 1.9 × 10^6^, 1.9 × 10^5^, 1.9 × 10^4^, 1.9 × 10^3^, 1.9 × 10^2^, 1.9 × 10^1^ copies per reaction; Lane 7: negative control; Gradient of purple bars: serially diluted DNA samples; Gray bar: negative control.

To determine the sensitivity of the *aadA1* LAMP assay, both LAMP and conventional PCR were performed using identical tenfold serial dilutions of genomic DNA. The LAMP assay consistently generated a positive signal with as little as 1 pg of DNA input per reaction (approximately 190 copies/reaction). This result was supported by the concurrent observation of a faint but distinct ladder-like band on agarose gel, a discernible shift to a blue color in the closed-tube system, and a measurable increase in the absorbance at 650 nm over the negative control (Figure 5A). In contrast, no visible band was detected by conventional PCR with 1 pg (190 copies) of DNA template (Figure 5B). These results demonstrate that the detection sensitivity of LAMP is 10-fold higher than that of PCR.

### 3.6. Clinical Application of a Visual LAMP Assay for the aadA1 Gene

To validate the optimized LAMP assay for *Salmonella aadA1* detection, 40 clinical isolates were tested simultaneously using the LAMP assay, the PCR, and the phenotypic tests with conventional bacterial cultures (Table 4). Based on the streptomycin resistance profile conferred by the *aadA1* gene, we classified 40 clinical *Salmonella* isolates into streptomycin-resistant and streptomycin-susceptible groups following antimicrobial susceptibility testing. Among these, 27 isolates (67.5%) were identified as streptomycin-resistant, while the remaining 13 were streptomycin-susceptible. Comparison of the LAMP and PCR methods against the phenotypic susceptibility data revealed that among the 27 resistant isolates, PCR detected the *aadA1* gene in 96.3% of the strains, whereas LAMP achieved a detection rate of 100.0%. Within the 13 susceptible isolates, PCR and LAMP produced positive rate in 23.1% and 38.5% of the strains, respectively. To resolve these discrepancies, all discordant samples—one resistant and five susceptible isolates—were subjected to Sanger sequencing. The results confirmed the presence of the *aadA1* gene in all six isolates. In summary, using sequencing as the reference method, the lower sensitivity of PCR relative to LAMP resulted in false-negative calls in a subset of *aadA1*-positive samples.

## 4. Discussion

Aminoglycoside antibiotics inhibit bacterial protein synthesis and are widely used as broad-spectrum agents against enteric bacterial infections [24,25]. Among them, streptomycin, kanamycin, and gentamicin are commonly deployed to treat Gram-negative pathogens such as *Salmonella*, *Shigella*, and *Proteus mirabilis*. However, the extensive use of these drugs has led to the emergence of antimicrobial resistance [26]. Of particular concern is the *aadA1* gene, a major determinant of streptomycin resistance that is frequently detected in *Salmonella* species [4]. Given its role in resistance dissemination, there is an urgent need to developed a convenient, rapid, and cost-effective method for *aadA1* detection.

In this study, we developed a closed-tube HNB-based LAMP assay for rapid detection of the *aadA1* gene. The reliability of such a visual detection system critically depends on the optimization of reaction components and conditions. Accordingly, systematic optimizations were conducted by testing gradients of MgSO_4_, dNTPs, Bst polymerase, primer ratios, as well as reaction temperature and duration. The optimal reaction conditions were determined by integrating the results of agarose gel electrophoresis, closed-tube HNB colorimetric analysis, and absorbance measurement. The colorimetric readout relies on the consumption of Mg^2+^ during amplification, which triggers a hydroxynaphthol blue (HNB)-mediated color shift from violet to sky blue [27,28]. Our systematic optimization revealed that MgSO_4_ concentration was the most critical factor for achieving a clear and reliable visual interpretation. While other LAMP studies report a wide range of MgSO_4_ concentrations (3–40 mM) [15,18,20,29,30,31], we found that concentrations of 2 and 4 mM caused a pre-amplification color change, confounding initial assessment, while a high concentration (12 mM) inhibited the reaction. Ultimately, we identified 6 mM as the optimal concentration, which provided the most distinct color contrast and robust amplification efficiency. This precise optimization ensures the assay’s readability and reliability, which are crucial for its application in resource-limited settings.

The LAMP assay developed in this study exhibited high specificity and sensitivity, with a detection limit of 1 pg (190 copies) of genomic DNA per reaction. This performance is similar with that of other LAMP-based methods reported in the literature. For example, Gong et al. [18] established an m-LAMP method detecting the *sul1*, *sul2*, and *sul3* genes with a limit of 0.5 pg; Mu et al. [32] reported a LAMP assay for the *msrA* gene with a sensitivity of 100 pg; and Qi et al. [20] developed a LAMP method for the *cfr* gene achieving 1 pg detection. Notably, the sensitivity of LAMP is generally 10- to 100-fold higher than that of conventional PCR.

Beyond its analytical performance, our closed-tube colorimetric LAMP assay offers substantial practical advantages over both conventional molecular methods and the existing LAMP alternative. When compared to the previously reported *aadA1* LAMP method that relies on gel electrophoresis [21], our approach is superior in its contamination control and field applicability. The closed-tube design with visual dye readout entirely eliminates the need for post-amplification processing, thereby preventing aerosol contamination and false positives, which are significant risks in open-tube systems. Furthermore, our LAMP protocol provides results in approximately 35 min through direct visual readout. This represents a significant reduction from the 2–3 h typically required for conventional PCR (including gel electrophoresis) and the 1.5–2 h needed for qPCR [10,33]. Critically, the isothermal nature of LAMP eliminates the need for the expensive thermal cyclers essential for both PCR and qPCR, requiring only a simple heating block. This combination of speed, minimal equipment dependency, intrinsic contamination control, and elimination of any post-amplification steps collectively underscores our assay’s strong potential as a robust tool for deployment in resource-limited settings and high-throughput screening scenarios.

The main limitation of the LAMP technique is its high susceptibility to aerosol contamination, which primarily occurs when opening reaction tubes after amplification—such as for gel electrophoresis. This risk arises from two factors: the extremely high amount of amplification products (often in the microgram range) generated during the isothermal process, and the formation of aerosol droplets carrying these products when tubes are opened. Even trace amounts of such contaminants can act as templates in future reactions, causing false-positive results and undermining assay reliability [34,35]. To minimize contamination risk, strict measures were implemented throughout this study. Amplification and post-amplification procedures were physically separated to prevent amplicon entry into clean reagent preparation areas. In addition, aerosol-resistant pipette tips were consistently used, gloves were changed frequently, and work surfaces were routinely decontaminated using a nucleic acid removal reagent (Labshark, Changde, China) combined with UV irradiation. Most importantly, the HNB-based closed-tube detection method developed in this study entirely eliminates the need to open tubes for result analysis, thereby substantially reducing the possibility of aerosol-mediated contamination.

This deliberate choice of HNB over other colorimetric indicators was pivotal to achieving this closed-tube format. Several alternative methods are available, including calcein/Mn^2+^ complexes and SYBR Green I [15,29,31,36]. However, calcein requires precise pre-mixing of multiple components, increasing preparation complexity, while SYBR Green I is mutagenic and must be added post-amplification, inevitably leading to the aerosol contamination risks that this study sought to avoid. In contrast, HNB is a single, safe reagent that can be pre-mixed into the reaction, enabling robust closed-tube detection and visual interpretation without compromising safety or simplicity. Therefore, HNB was selected as the optimal indicator to align with our goal of developing a robust, field-deployable assay.

To evaluate the clinical applicability of the LAMP assay, we compared its performance against PCR and phenotypic susceptibility testing using 40 clinical *Salmonella* isolates. Phenotypic analysis indicated a streptomycin resistance rate of 67.5%, consistent with previous reports [5,37]. Among the resistant isolates, PCR detected the *aadA1* gene in 96.3% of strains, while LAMP achieved 100.0% detection. All cases with discrepant results were confirmed by sequencing to harbor *aadA1*, demonstrating the superior sensitivity of LAMP. Among streptomycin-sensitive isolates, *aadA1* was detected in 23.1% of strains by PCR and 38.5% by LAMP, revealing a notable genotype–phenotype discrepancy. To elucidate the mechanisms underlying this discordance, we sequenced the *aadA1* gene from these sensitive yet gene-positive isolates. While nucleotide polymorphisms were identified at positions 11 (T/C), 331 (A/G), 602 (G/A), and 750 (C/T), their role in conferring the sensitive phenotype appears limited. Critically, at each polymorphic site, both nucleotide variants were found among the sensitive isolates, and no uniform, inactivating mutation was common to all. This finding suggests that these polymorphisms likely represent natural sequence variation rather than being the primary cause of protein inactivation. Therefore, while mutations remain a theoretical possibility, our data do not support them as the dominant mechanism in our strain set. Consequently, the observed discrepancy is more likely attributable to the following mechanisms: (i) Transcriptional silencing or low expression of the *aadA1* gene, resulting in insufficient protein production to confer resistance [38,39]; (ii) Streptomycin resistance is often multifactorial, involving combinations of enzymatic modification, efflux pumps, or target alterations; thus, the presence of *aadA1* alone may be insufficient to produce a resistant phenotype [4,39,40]; (iii) The high sensitivity of molecular methods capable of detecting low-copy-number genes that do not translate to a clinical resistance phenotype; (iv) phenotypic susceptibility is determined by zone diameter standards; although an inhibition zone is present, strains are classified as susceptible as long as the zone diameter does not fall below the defined resistance breakpoint [41]. These results indicate that molecular methods such as LAMP and PCR are valuable as early-warning systems for identifying resistance gene reservoirs and transmission risks. However, their results should be interpreted in context, and integration of genotypic and phenotypic data remains essential for accurately assessing antimicrobial resistance dynamics.

## 5. Conclusions

The optimized visual LAMP assay for detecting the *Salmonella aadA1* gene demonstrated high specificity and sensitivity, with a detection limit of 1 pg (190 copies) of genomic DNA. The method’s combination of rapid isothermal amplification (within 35 min) and minimal equipment requirements makes it particularly suited for resource-limited settings. This provides a practical tool for grassroots laboratories, enabling local-scale detection critical for understanding and curbing the spread of streptomycin resistance. Future work will focus on validating the assay’s performance with complex food and environmental samples to assess its field robustness.

## Figures and Tables

**Table 1:** The *Salmonella* reference strains used in this study.

NO.	Strains	Serotype	*aadA1*
1	LLC-35	*S. pullorum*	+
2	LLC-41	*S. pullorum*	+
3	LLC-51	*S. pullorum*	+
4	HO-43	*S. Newport*	+
5	HO-96	*S. pullorum*	+
6	HO-78	*S. pullorum*	−
7	LLC-17	*S. enteritidis*	−
8	LLC-19	*S. pullorum*	−
9	LLC-8	*S. Newport*	−
10	BSC-16	*S. enteritidis*	−

**Table 2:** Primer sequences for *aadA1* gene for LAMP and PCR.

Method	Primers	Sequence (5′–3′)	References
LAMP	F3	GTTGTGCACGACGACATCA	This study
	B3	GGATCAAAGAGTTCCTCCGC	
	FIP	TGCAAGAATGTCATTGCGCTGCGTGGCGTTATCCAGCTAAGC	
	BIP	GGTATCTTCGAGCCAGCCACGCCAAGGCAACGCTATGTTCT	
	LB	TCTGGCTATCTTGCTGACAAAAGC	
PCR	*aadA1*-F	TATCCAGCTAAGCGCGAACT	[1]
	*aadA1*-R	ATTTGCCGACTACCTTGGTG	

**Table 3:** Optimized parameters for *Salmonella aadA1* LAMP Reaction.

Parameters	Assay Range	Optimized
Isothermal Amplification Buffer	1×	1×
MgSO_4_	2–12 mM	6 mM
dNTPs	0.2–1.4 mM	1.4 mM
Bst Polymerase	1.6–16 U	8 U
FIP/BIP Primers	0.2–2.8 µM	1.6 µM
F3/B3 Primers	0.2 µM	0.2 µM
Loop F Primers	0.4 µM	0.4 µM
Nuclease-Free H_2_O	Make up to 25 µL	Make up to 25 µL
Betaine	1 M	1 M
DNA Template	1 µL	1 µL
Hydroxynaphthol blue	120–240 μmol/L	120 μmol/L
Temperature	58–66 °C	64 °C
Time	25–55 min	35 min

**Table 4:** Comparison of the optimized LAMP and PCR assays with phenotypic testing for the detection of *aadA1* in clinical *Salmonella* isolates.

Phenotypic Tests	LAMP		PCR		Total
	+	−	+	−	
Streptomycin-resistant	27 (100.0%)	0	26 (96.3%)	1 (3.7%)	27
Streptomycin-susceptible	5 (38.5%)	8 (61.5%)	3 (23.1%)	10 (76.9%)	13
Total	32 (75.0%)	8 (25.0%)	29 (72.5%)	11 (27.5%)	40

## Data Availability

The original contributions presented in this study are included in the article. Further inquiries can be directed to the corresponding author.
